# SEOM clinical guideline for management of adult medulloblastoma (2020)

**DOI:** 10.1007/s12094-021-02581-1

**Published:** 2021-04-01

**Authors:** R. Luque, M. Benavides, S. del Barco, L. Egaña, J. García-Gómez, M. Martínez-García, P. Pérez-Segura, E. Pineda, J. M. Sepúlveda, M. Vieito

**Affiliations:** 1 Department of Medical Oncology, Hospital Universitario Virgen de Las Nieves, Granada, Spain; 2 Department of Medical Oncology, Hospital Regional Universitario Carlos Haya, Malaga, Spain; 3 Department of Medical Oncology, Hospital Universitari Dr. Josep Trueta. ICO Girona, Girona, Spain; 4 Department of Medical Oncology, Hospital Donostia-Donostia Ospitalea, San Sebastián, Spain; 5 Department of Medical Oncology, Complexo Hospitalario de Ourense (CHUO), Orense, Spain; 6 Department of Medical Oncology, Hospital del Mar - Parc de Salut Mar, Barcelona, Spain; 7 Department of Medical Oncology, Hospital Universitario Clínico San Carlos, Madrid, Spain; 8 Department of Medical Oncology, Hospital Clinic I Provincial de Barcelona, Barcelona, Spain; 9 Department of Medical Oncology, Hospital Universitario 12 de Octubre, Madrid, Spain; 10 Department of Medical Oncology, Hospital Universitario Vall D’Hebron, Barcelona, Spain

**Keywords:** Medulloblastoma, Adults, Treatment

## Abstract

Recent advances in molecular profiling, have reclassified medulloblastoma, an undifferentiated tumor of the posterior fossa, in at least four diseases, each one with differences in prognosis, epidemiology and sensibility to different treatments. The recommended management of a lesion with radiological characteristics suggestive of MB includes maximum safe resection followed by a post-surgical MR < 48 h, LCR cytology and MR of the neuroaxis. Prognostic factors, such as presence of a residual tumor volume > 1.5 cm^2^, presence of micro- or macroscopic dissemination, and age > 3 years as well as pathological (presence of anaplastic or large cell features) and molecular findings (group, 4, 3 or p53 SHH mutated subgroup) determine the risk of relapse and should guide adjuvant management. Although there is evidence that both high-risk patients and to a lesser degree, standard-risk patients benefit from adjuvant craneoespinal radiation followed by consolidation chemotherapy, tolerability is a concern in adult patients, leading invariably to dose reductions. Treatment after relapse is to be considered palliative and inclusion on clinical trials, focusing on the molecular alterations that define each subgroup, should be encouraged. Selected patients can benefit from surgical rescue or targeted radiation or high-dose chemotherapy followed by autologous self-transplant. Even in patients that are cured by chemorradiation presence of significant sequelae is common and patients must undergo lifelong follow-up.

## Methodology

This guideline has been developed based on the consensus of ten medical oncologists, designated by the Spanish Society of Medical Oncology (SEOM) and the Spanish Neuro-Oncology Research Group (GEINO), with the purpose of reviewing and summarizing the available evidence regarding the management of meduloblastoma (MB), as well as generating evidence-based statements on diagnostics and therapeutic strategies. To be in accordance with previous SEOM guidelines, the rating system for quality of the evidence (I–III) and strength of the recommendation (A–E) criteria are summarized in Table 1.

**Table 1:**
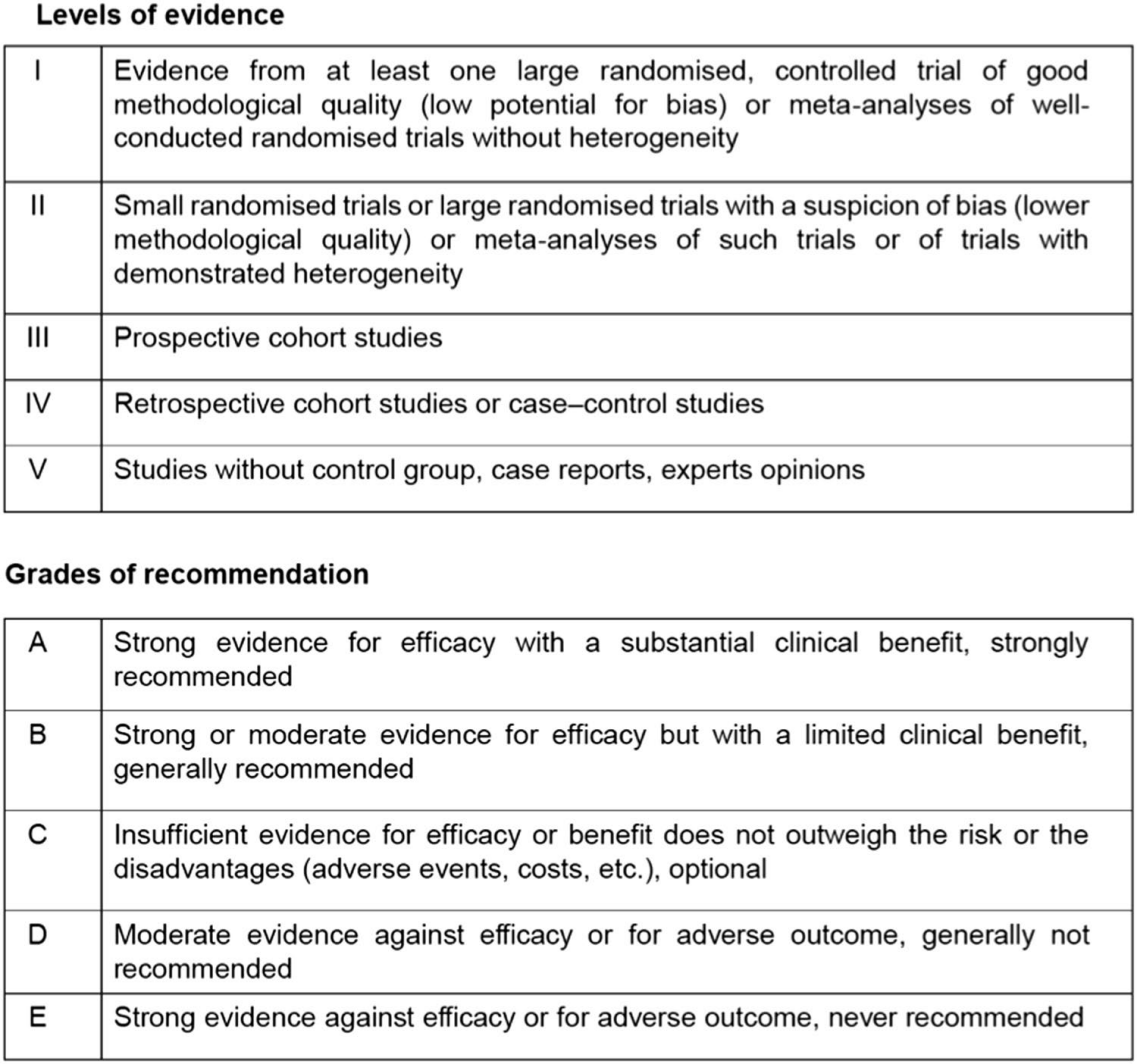
Levels of evidence

## Introduction

Among the more than 100 different types of primary central nervous system (CNS) tumors, MB, sometimes referred as primitive neuroectodermal tumor of the posterior fossa [1] is a subtype of embryonal tumor. Due to its high proliferative index and risk of metastatization thorough the CNS following CSF pathways, it is classified by WHO as grade IV tumor.

The four long-established histological variants (classic, desmoplastic/nodular, extensive nodularity, large cell/anaplastic) have been more recently genetically defined in four different molecular which have divergent genetics, clinical behavior, and patient outcomes and can be used for an integrated diagnosis [2].

### Epidemiology

Consistently with its embryonal origin, MB is much more common in childhood, and extremely rare (about 1% of primary CNS tumors) in adults.

The majority of medulloblastomas are sporadic but some cases might be traced to a hereditary cancer syndrome, namely Turcot, Li Fraumeni and Gorlin syndrome in SHH MB and familiar polyposis coloni in WNT MB [3].

Although MBs are potentially curable tumors by applying a multimodal treatment approach, the survival is largely influenced by age (adults worse), the extent of the disease, residual disease after resection and the molecular subtype.

## Histology and molecular biology

As most embryonal tumors, MB displays diffuse and high cellularity. Cells are small, poor-differentiated, round or oval, forming diffuse masses or, sometimes well-defined nodes. Rossettes are typical structures of medulloblastomas and are characterized by groups of cells set in a circle with a fibrillary center. Although they typically remain undifferentiated, some MBs contain cells with neuronal or glial differentiation.

Immunohistochemical staining frequently demonstrates expression of neuronal markers, such as synaptophysin and enolase, and also embryonal or neuroepithelial markers, such as nestin. Other immunohistochemistry studies are important to differentiate the subgroups, in fact, MB diagnosis must integrate morphological and molecular features.

The last WHO classification of the tumors of the CNS (2016) differentiates four sub-types[1] that are summarized in Table 2:

1. WNT-activated medulloblastoma:

This subgroup is the most uncommon type of MB and represents about 10% of cases. WNT-activated MBs are very rare in infants but can be seen in adults or children.

The Wnt protein can deactivate the protein complex that eliminates Beta-catenin. The activation of this pathway turns into an accumulation of beta-catenin in the nucleus where it acts as a transcription factor of proliferation genes, such as *Cyclin D* and c-*myc*.

This subgroup of MB has the better prognosis in children, with a five-year overall survival of 95%. In adults, the prognosis is also good but the 5-year overall survival is below 95% [4].

2. SHH-activated MB:

The sonic-hedgehog pathway is important in the proliferation of the cerebellar Purkinje cells. SHH pathway activation is present in 30% of MBs and most of them are histologically desmoplastic. *TP53* mutations have been found in 10–20% of this subgroup and this molecular alteration has important prognostic consequences since the five-year overall survival rate is approximately 40% while the TP53wt SHH-activated MBs have a five-year survival rate of 80%. SHH-activated MBs with *TP53* mutations have a peak incidence in adolescence while *TP53*wt SHH are more common in infants and adults. It is possible that in further classifications SHH-activated MBs will be split within two independent groups depending on T*P53*.

3. Group 3:

This group is characterized by the presence of high-level *MYC* amplification and significant genomic instability and other alterations (see Table 2). This group (25% of MB) is very rare in adults, but not in children. Morphologically, most of the cases are large cell/anaplastic variant. The prognosis is poor with frequent metastases at diagnosis and a 5-year survival rate of only 50% [5].

**Table 2:** Molecular subgroups of MB according to WHO 2016

	WNT-activated	SHH-activated	Group 3	Group 4
Gene expression and genetics	WNT signaling *CTNNB1* mutation	SHH signaling: *PTCH11/SMO/SUFU* mutation *GLI2* amp TP53 mut *MYCN* amplification	*MYC* amplification *SMARCA4* mutation *OTX2* amplification GFI I enhancer activation	*MYCN* amplification *CDK6* amplification
Demographics	Children > Adults Rarely in Infants	Infants and adults	Infants > children Rarely in adults More common in male	Children Possible in infants and adults More common in male
Outcome	Very good 5-year OS > 95%	Infants: good Others: Intermediate *TP53*mut worse prognosis	Very poor	Intermediate Adults: poor
Histology	Classic	Desmoplasic/nodular	Classic large cell/anaplastic	Classic
Risk of metastases	Low	Low	High	High

4. Group 4:

This group comprises 35% of MBs and its main feature is the amplification of both *MYCN* and cyclin-dependent kinase 6 (*CDK6*) genes [6]. The histology uses to be the classic pattern. Group 4 tumors are more common in males with a peak incidence in adolescence. The prognosis is different in children and adults, while the prognosis is similar to the SHH group in children, five-year survival rate of 75%, in adults is worse, even with the large cell/anaplastic morphology [1].

## Diagnosis and staging

The clinical symptoms and signs are associated with the location of the tumor in the posterior fossa, such as increased intracranial pressure, cerebellar dysfunction and/or obstruction of cerebrospinal fluid pathway. Symptoms evolve over a period of weeks to a few months. However, adult MB may have an indolent clinical course that could be in the range 1–18 months (mean 7 months). Thus, MB in adult patients sometimes debuts insidiously, with headache, dizziness, nausea, ipsilateral cerebellar signs, and ataxia [7].

In general, MB arises from the cerebellum and the floor of the fourth ventricle. In adult MB, the origin in lateral cerebellar hemispheres or cerebellopontine angle predominates over midline structures, and cyst formation is more common. MRI of the brain demonstrates iso- to hypointense T1 and hyper- to hypointense T2 lesions. MBs typically show a characteristic diffusion restriction with increased signal on diffusion-weighted imaging (DWI) sequence corresponding to decreased signal on apparent diffusion coefficient (ADC) sequence. However, adult MB lesions are more likely to demonstrate inhomogeneous contrast enhancement, hyperintense signal on T1, and hypointense signal on T2 sequences and are less likely to demonstrate contrast enhancement when compared with pediatric MB lesions. These differences in MRI appearance may be explained by substantial molecular differences that may influence neuroradiologic findings.

All newly diagnosed MB patients should undergo staging including postoperative MRI of the brain (within 48 h after surgery), MRI of the entire spine with and without contrast (it should be delayed by at least 2–3 weeks post surgery to avoid post-surgical artifacts) and lumbar puncture with cytology (it should be done after spine MRI and should be delayed at least 2 weeks after surgery to avoid possible false-positive cytology). The neurological exam and degree of suspicion for spinal spread will determine the number of times lumbar puncture should be repeated to increase the diagnostic yield (level IIA) [8].

Systemic staging is warranted if there are symptoms or signs of extra-CNS involvement (Bone scan; CT with contrast of chest, abdomen, and pelvis or whole body PET/CT and bone marrow biopsy only if clinically indicated) (level IIA) [8].

Clinical staging has been used for risk stratification and to determine the intensity of adjuvant treatment. Chang staging takes tumor size and disease spread to determine low- vs. high-risk group. Packer is more used in both adults and children (Table 3) [9].

**Table 3:** Packer staging criteria for Medulloblastoma

High risk	Standard risk
Residual disease > 1.5 cm^2^	Residual disease < 1.5 cm^2^AND
Disseminated disease (M1-M4) OR	No metastasis (M0) AND
Age < 3 years	Age > 3 years

## Prognostic factors

Historically, risk stratification factors for MB are based on clinical features, such as age, extent of disease at diagnosis, extent of surgical resection and tumor histology. So generally, standard risk (SR) is defined as having residual tumor less than 1.5 cm^2^, no metastatic disease and classic or desmoplastic histology. High risk (HR) includes all other patients. Additional studies have emphasized the importance of both molecular markers and histopathology in determining prognosis, and these now form the basis for better pretreatment risk stratification [10].

MB is rare in adults, and there are no randomized trials which to base treatment recommendations or analyzing prognostic factors. There are conflicting data on the survival rates of adults with MB compared to those of children. In a recent population-based study using Surveillance, Epidemiology, and End Results database that included children and adults with MB treated between 1992 and 2013, 5-year and 10-year survival rates were 75.5%, 74.2%, 67.9% and 67.3%, respectively. Multivariable regression modeling found that gross total resection, radiotherapy treatment was associated with a better prognosis. Large cell/anaplastic histology was associated with poor prognosis [11].

## Multidisciplinary management

Therapy of adult MB is mostly based on pediatric studies and only small retrospective and few prospective adult trials are available [12]. Standard treatment comprises a combination of maximal safe resection, craniospinal irradiation (CSI), and chemotherapy (CT). A molecular classification of MB is starting to translate into clinics, and subgroup-specific approaches will ideally allow an accurate selection of radiation dosage or CT schedules, and specific targeted therapies [13]. However, the standard of care is still based on clinical classification.

### Surgery

The first step of multimodal treatment is maximal safe resection. The prognostic relevance of extent of resection (EOR) in MB is still controversial. In pediatric population, EOR did not showed a significant survival benefit for WNT, SHH, or group 3. However, there was a progression-free survival (PFS) benefit in group 4. None of these studies have found an association between EOR and overall (OS). Evidence suggests that maximal safe resection should remain the aim of initial surgery [14] [Level III B].

### Radiation therapy

The next step in the therapy for both standard-risk (SR) and high-risk (HR) patients is radiation therapy (RT), and it is commonly delivered as CSI with a boost to posterior fossa or the tumor bed [15] [Level I A]. RT represents a favorable prognostic factor in adult patients. Worse outcomes have been reported when RT was delayed more than 3–6 weeks after surgery [16]. Evidence from pediatric prospective randomized trials showed that a shorter time to completion of RT was associated with improved event-free survival (EFS). We recommend that RT should start 3–6 weeks after surgery with minimal disruptions.

RT dose in adults is not well established. These patients can experience acute side effects (hematologic and gastrointestinal for example) and also frequent neurocognitive deterioration. CSI is usually delivered with full dose (36 Gy) with a boost of 18.8 Gy to posterior fossa (up to 54–55.8 Gy) in addition to CT (17). RT alone is an option for unfit patients. In SR adult patients, dose-reduced CSI (23.4 Gy) in combination with CT is still under study, but it could be used based on pediatric studies (level IIIB). There are trials ongoing.

Another strategy to ameliorate toxicity of CSI could be proton beam therapy [18] [Level III B]. A decrease in hematologic toxicity would facilitate the use of chemotherapy in adults but there is no prospective evidence, and proton radiation is not widely available yet.

### Systemic therapy

Chemotherapy (CT) recommendations for adult patients suffer from a lack of randomized studies. Treatment recommendations are based on retrospective analysis of adult cohorts within pediatric trials, few single arm adult studies and a meta-analysis.

In HR patients, the available evidence suggests that adjuvant CT is associated with improved survival compared with RT alone.

The Packer regimen consisting of eight doses of vincristine during RT, followed by eight cycles of lomustine, cisplatin and vincristine administered in 6-week cycles [15] is the most used protocol, but dose modifications are required in nearly all adult patients. The prospective single arm phase II NOA-07 trial evaluated feasibility and toxicity of Packer regimen in 33 patients older than 21. 70% of patients tolerated at least 4 cycles of chemotherapy, all of them with dose modifications. The 3-year event-free survival rate was 66.6%, and the 3-year overall survival rates were 70%. In the prospective analysis of adults over 21y with non-metastatic MB who were not meeting the inclusion criteria for the pediatric trial HIT 2000, 47 of 49 patients required dose modifications. The 4-year event-free and overall survival rates were 68 and 89%, respectively. To avoid the dose reductions caused by hematologic toxicity after radiation, some propose the use of CT pre-radiation. A non-comparative phase II study by Brandes et al., in which HR patients received chemotherapy before RT and low-risk patients RT alone. No significant difference in PFS and OS was observed [12].

In SR patients, it is less clear than in children, but a recent meta-analysis showed that chemotherapy (neo or adjuvant) given as first-line significantly improved survival and increased the chance for long-term survival in all patients [19].

We recommend adult patients are treated with CT in first line, in addition to surgery and radiotherapy, irrespective of their risk profile. [level IIA].

## Relapse and metastatic disease. Therapy in Relapse

There are no standard treatments for recurrent MB and prognosis is very poor, less than 12 months in most cases. So, inclusion of these patients in clinical trials should be encouraged.

Second surgery must be considered when a complete resection can be achieved, and for palliative relief of symptoms (level IIIA).

Re-irradiation, especially focal radiotherapy, such as stereotactic radiotherapy or radiosurgery, can be considered as a possible and safe treatment in selected cases of focal recurrent MB relapse. Radiobiological aspects should be considered (level III B) [20].

Systemic treatment also could be an option especially in multifocal relapses. Different regimens have demonstrated some benefit in children with recurrent MB, and can be used in adults considering the age-specific biology (level IIIB) [8]:

- Metronomic anti-angiogenic therapy: MEMMAT schema: bevacizumab, thalidomide, celecoxib, fenofibrate, etoposide and cyclophosphamide, and additional intraventricular treatment; Kieran protocol: etoposide, cyclophosphamide,thalidomide, celecoxib and fenofibrate,
- Platinum—etoposide-based chemotherapy
- Temozolomide monotherapy [28]
- Temozolomide combinations: TOTEM (topotecan-temozolomide) or TEMIRI (temozolomide-irinotecan ± bevacizumab).

High-dose chemotherapy (HDC) with autologous stem cell transplantation (ASCT) has been explored in small series in selected centers. In a single-institution study, the outcomes of adult patients treated with HDC with ASCT compared with a cohort of patients who received conventional-dose chemotherapy (CDC) show increase in survival from 2 years to 3.47 years. Conditioning regimens employed with ASCT were thiotepa with carmustine, carboplatin (± ethoposide) [21]. Other conditions of regimens that have been reported associated with increased survival include carboplatin thiotepa and etoposide followed by ASCR (EFS/OS at 10y 24%) [22].

**Fig. 1:**
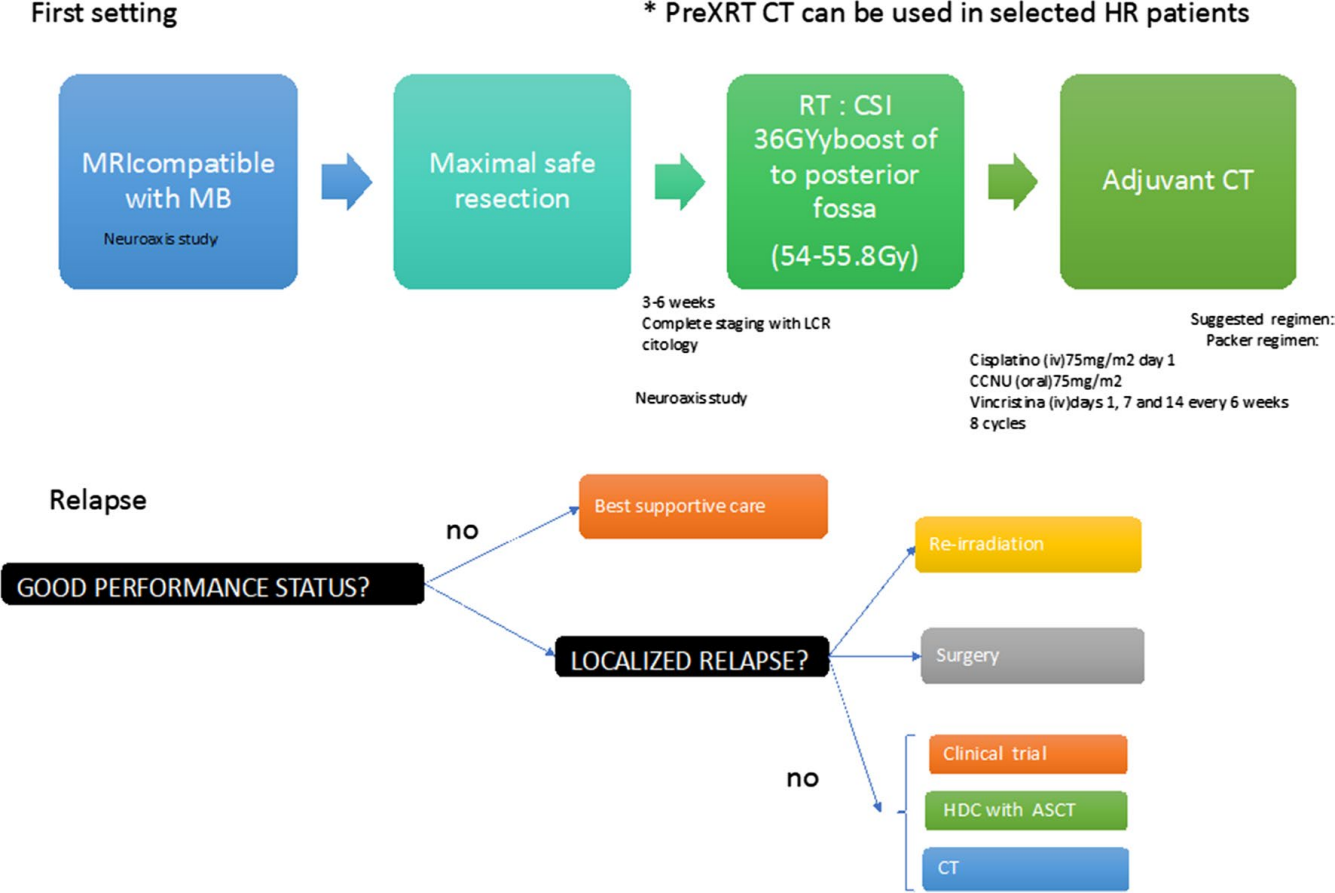
Therapeutic algorithm

Due to the risk of severe toxicity, especially in patients that have received craniospinal radiation and even toxic deaths and the lack of prospective evidence, this option should only be considered in selected patients (level III B) (Fig. 1).

## Targeted therapy and investigational strategies

SHH MBs, the majority subgroup in adults, are characterized by the abnormal activation of the Sonic-Hedgehog pathway. Despite the disappointing results of early clinical trials with SMOi inhibitors, a retrospective review shows that most of the adult patients, who present upstream alterations respond to SMO inhibition while the pediatric patients mostly present downstream alterations, such as SUFU mutations of GLI amplifications, which are intrinsically resistant [23].

For those patients, preclinical studies point to the possibility of interfering with GLI, either by interfering with GLI translation, using arsenic trioxide compounds or by interfering with GLI-induced expression changes, for example, using Bet inhibitors [24].

The WNT subgroup is characterized by the presence of catenin B mutations in up to 80% of cases. This subgroup is the less frequent in adults in which only comprises 10% of cases. Due to their good prognosis, the prevalence in the refractory setting is very low and personalized treatment strategies have focused on trying to deescalate certain treatments to minimize toxicity in this subgroup. However, the co-existence of alterations on the SWI/SNF complex could open the opportunity to personalized treatment with epigenetic modulators, such as PRC2 inhibitors [25].

Non-SHH-non WNT MBs in adults almost inevitably correspond to the group 4 MB, and are a genetically heterogeneous subgroup that is more characterized by structural changes, such as the presence of SNCAIP duplications, that by mutations in specific pathways, although a significant minority have alterations the NOTCH and TGFB pathway. However, the presence of a MYC amplification is a potential targetable event in this subgroup, as are in a minority of cases, the presence of HRD-associated genes, such as BRCA, PALB2 and FANCA [26].

## Side effects, follow-up and patient care

### Side effects

Side effects are age-dependent, with the elderly being more prone to toxicities secondary to surgery and the younger to other therapies.

Surgery: they are due to the involvement of the stem, fundamentally. Posterior fossa syndrome occurs in up to 25% of cases. It seems that the association of radiotherapy may increase the incidence of this condition.

Radiotherapy: acute effects are anorexia and nausea as well as nutritional deterioration. In the long term, these effects are more important in young people and focus mainly on cognitive decline, risk of developing second radio-induced tumors (especially glioblastoma and meningioma). Other toxicities include endocrinopathies, cardiac and pulmonary toxicity, ototoxicity, and vascular toxicity.

Chemotherapy: Vincristine-induced peripheral neuropathy, which can be as high as 37%. As for cisplatin, it can cause ototoxicity (34% incidence with 9% G3-4) and myelosuppression, mainly [27].

### Follow-up

Late relapses are possible, especially in adults. Brain MRI every 3 months for the first 2 years; then every 6–12 months until they are 5–10 years old; and subsequently every 1–2 years.

In those patients with spinal involvement, spinal MRI should be added with the same frequency [8].

### Patient care

Although the quality-of-life aspects secondary to the type of tumor and the treatments received are important, it has been seen that there are subjective aspects that impact in the same way and that we must take into account. A multidisciplinary approach including psychosocial support, management of treatment sequelae, such as endocrinopathies and screening of treatment-induced cancer, is integral part of MB treatment [28].

## Conclusion

The management of MB in adults has to strike a balance between acknowledging the differences between adults and children, differences that influence MB biology, but also the ability of the patient to receive local and systemic treatment and different psychosocial backgrounds, and make use of the similarities to translate the available evidence that has been generated mostly by pediatric trials.

Due to the extreme rarity and high molecular heterogeneity of adult MB, it is almost impossible to make recommendations based on high levels of evidence, rather, the current guidelines and others recently published, such as the EANO-EURACAN guideline (17), should be used as a starting point and tailored to the specific age, performance status and comorbidities of the patient.

## References

[1] Louis DN, Perry A, Reifenberger G (2016). The 2016 World Health Organization classification of tumors of the central nervous system: a summary. ActaNeuropathol.

[2] Northcott PA, Korshunov A, Witt H (2011). Medulloblastoma comprises four distinct molecular variants. J ClinOncol.

[3] Waszak SM, Northcott PA, Buchhalter I (2018). Spectrum and prevalence of genetic predisposition in medulloblastoma: a retrospective genetic study and prospective validation in a clinical trial cohort. Lancet Oncol.

[4] Zhao F, Ohgaki H, Xu L (2016). Molecular subgroups of adult medulloblastoma: a long-term single-institution study. Neuro Oncol.

[5] Kool M, Korshunov A, Remke M (2012). Molecular subgroups of medulloblastoma: an international meta-analysis of transcriptome, genetic aberrations, and clinical data of WNT, SHH, Group 3, and Group 4 medulloblastomas. ActaNeuropathol.

[6] Northcott PA, Buchhalter I, Morrissy AS (2017). The whole-genome landscape of medulloblastoma subtypes. Nature.

[7] Majd N, Penas-Prado M (2019). Updates on management of adult medulloblastoma. Curr Treat Options Oncol.

[8] NCCN Clinical Practice Guidelines in Oncology (NCCN Guidelines®) Central Nervous System Cancers Version 2.2020—April 30, 2020

[9] Packer RJ, Goldwein J, Nicholson HS (1999). Treatment of children with medulloblastomas with reduced-dose craniospinal radiation therapy and adjuvant chemotherapy: a Children’s. Cancer Group Study J ClinOncol.

[10] Schwalbe EC, Lindsey JC, Nakjang S (2017). Novel Molecular Subgroups for clinical classification and outcome prediction in childhood medulloblastoma: a cohort study. Lancet Oncol.

[11] Li Q, Dai Z, Cao Y, Wang L (2018). Comparing children and adults with medullloblastoma: a SEER based analysis. Oncotarget.

[12] Brandes AA, Franceschi E, Tosoni A, Blatt V, Ermani M (2007). Long-term results of a prospective study on the treatment of medulloblastoma in adults. Cancer.

[13] Thompson MC, Fuller C, Hogg TL (2006). Genomics identifies medulloblastoma subgroups that are enriched for specific genetic alterations. J ClinOncol.

[14] Thompson EM, Bramall A, Herndon JE et al. The clinical importance medulloblastoma extent of resection: a systematic review, Vol. 139, Journal of Neuro-Oncology. Springer New York LLC; 2018. p. 523–39. 10.1007/s11060-018-2906-5 29796724

[15] Packer RJ, Gajjar A, Vezina G (2006). Phase III study of craniospinal radiation therapy followed by adjuvant chemotherapy for newly diagnosed average-risk medulloblastoma. J ClinOncol.

[16] Abacioglu U, Uzel O, Sengoz M (2002). Medulloblastoma in adults: treatment results and prognostic factors. Int J RadiatOncolBiol Phys.

[17] Franceschi E, Hofer S, Brandes AA (2019). EANO–EURACAN clinical practice guideline for diagnosis, treatment, and follow-up of post-pubertal and adult patients with medulloblastoma. Lancet Oncol.

[18] Brown AP, Barney CL, Grosshans DR (2013). Proton beam craniospinal irradiation reduces acute toxicity for adults with medulloblastoma. Int J RadiatOncolBiol Phys.

[19] Kocakaya S, Beier CP, Beier D (2016). Chemotherapy increases long-term survival in patients with adult medulloblastoma–a literature-based meta-analysis. Neuro Oncol.

[20] Buglione M, Triggiani L, Grisanti S (2013). Retreatment of recurrent adult medulloblastoma with radiotherapy: a case report and review of the literature. J Med Case Rep.

[21] Gill P, Litzow M, Buckner J (2008). High-dose chemotherapy with autologous stem cell transplantation in adults with recurrent embryonal tumors of the central nervous system. Cancer.

[22] Dunkel IJ, Gardner SL, Garvin JH (2010). High-dose carboplatin, thiotepa, and etoposide with autologous stem cell rescue for patients with previously irradiated recurrent medulloblastoma. Neuro Oncol.

[23] Robinson GW, Orr BA, Wu G (2015). Vismodegib exerts targeted efficacy against recurrent sonic hedgehog—subgroup medulloblastoma: Results from phase II Pediatric Brain Tumor Consortium studies PBTC-025B and PBTC-032. J ClinOncol.

[24] El Doussouki M, Gajjar A, Chamdine O (2019). Molecular genetics of medulloblastoma in children: Diagnostic, therapeutic and prognostic implications. Future Neurol.

[25] Sengupta S, Pomeranz Krummel D and Pomeroy S. The evolution of medulloblastoma therapy to personalized medicine [version 1; peer review: 3 approved]. F1000Research 2017, 6(F1000 Faculty Rev):490. 10.12688/f1000research.10859.1 10.12688/f1000research.10859.1 PMC5490254 28713553

[26] Menyhárt O, Giangaspero F, Gyorffy B (2019). Molecular markers and potential therapeutic targets in non-WNT/non-SHH (group 3 and group 4) medulloblastomas. J HematolOncol.

[27] Thomas A, Noël G (2019). Medulloblastoma: Optimizing care with a multidisciplinary approach. J MultidiscipHealthc.

[28] Giovagnoli AR, Meneses RF, Silvani A (2014). Quality of life and brain tumors: What beyond the clinical burden?. J Neurol.

